# Supplementary material for: Causal Relationship between Aging and Anorexia Nervosa: A White-Matter-Microstructure-Mediated Mendelian Randomization Analysis
Source: Biomedicines. 2024 Aug 16;12(8):1874. doi: 10.3390/biomedicines12081874 (PMC11351342; doi:10.3390/biomedicines12081874)
Supplement: Supplementary file 1 [file biomedicines-12-01874-s001.zip › Supplementary Figure/Figure S8/Leaveoneout plot of MR analysis for TL to ubm-b-1993.pdf]

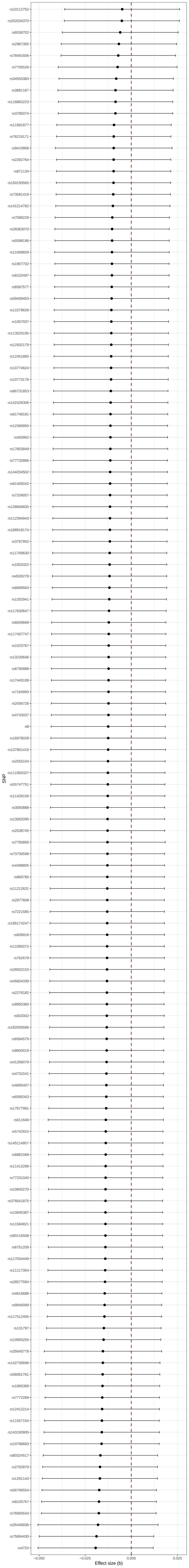

rs10112752
rs202034370
rs6536702
rs2967355
rs78491606
rs7705526
rs34550083
rs3891167
rs116863223
rs3785074
rs11991877
rs76219171
rs9419958
rs2282764
rs871134
rs150150565
rs73581419
rs141214782
rs7099229
rs28363070
rs9398196
rs11699829
rs1907702
rs8102497
rs6587577
rs59409453
rs11579626
rs1957937
rs113525195
rs12932179
rs12451892
rs10774624
rs10773176
rs66731853
rs142426306
rs61748181
rs12369950
rs450962
rs17803849
rs77732866
rs144204502
rs61405042
rs7209057
rs139669835
rs112394943
rs188918174
rs3767952
rs11769630
rs1003322
rs4530278
rs6669563
rs1332941
rs117630647
rs6659669
rs117407747
rs1023767
rs13230646
rs6790988
rs17445108
rs7164950
rs2056726
rs4743037
All
rs16978028
rs137901416
rs2555104
rs111950327
rs55747751
rs11426156
rs3093888
rs13062095
rs2538745
rs7790856
rs73730598
rs4498805
rs869785
rs11212631
rs2977608
rs7221585
rs185174247
rs939916
rs11085072
rs762679
rs28502153
rs45604339
rs2276182
rs9955360
rs932002
rs182059586
rs6584579
rs9600019
rs41269078
rs4731541
rs4695407
rs6590343
rs17677991
rs611646
rs5742915
rs145114957
rs6881568
rs11412296
rs77231040
rs10840270
rs376641875
rs10845387
rs11584821
rs80116508
rs6751209
rs117034449
rs11117354
rs28577594
rs4616688
rs9940099
rs117512405
rs131797
rs10905255
rs35640778
rs142730696
rs56061761
rs1985369
rs7772289
rs12412214
rs11557154
rs143190905
rs10768683
rs80324517
rs2763979
rs1291143
rs56799554
rs8105767
rs76065543
rs35446936
rs75684430
rs4724
SNP
-0.050
-0.025
0.000
0.025
Effect size (b)
